# Loss of SNHG4 Attenuated Spinal Nerve Ligation-Triggered Neuropathic Pain through Sponging miR-423-5p

**DOI:** 10.1155/2020/2094948

**Published:** 2020-05-06

**Authors:** Xia Pan, Cheng Shen, Yayi Huang, Long Wang, Zhongyuan Xia

**Affiliations:** Department of Anesthesia, Renmin Hospital of Wuhan University, Wuhan Hubei, China

## Abstract

Neuropathic pain is an intractable comorbidity of spinal cord injury. Increasing noncoding RNAs have been implicated in neuropathic pain development. lncRNAs have been recognized as significant regulators of neuropathic pain. lncRNA Small Nucleolar RNA Host Gene 4 (SNHG4) is associated with several tumors. However, the molecular mechanisms of SNHG4 in neuropathic pain remain barely documented. Here, we evaluated the function of SNHG4 in spinal nerve ligation (SNL) rat models. We observed that SNHG4 was significantly upregulated in SNL rat. Knockdown of SNHG4 was able to attenuate neuropathic pain progression via regulating behaviors of neuropathic pain including mechanical and thermal hyperalgesia. Moreover, knockdown of SNHG4 could repress the neuroinflammation via inhibiting IL-6, IL-12, and TNF-*α* while inducing IL-10 levels. Additionally, miR-423-5p was predicted as the target of SNHG4 by employing bioinformatics analysis. miR-423-5p has been reported to exert significantly poorer in several diseases. However, the role of miR-423-5p in the development of neuropathic pain is needed to be clarified. Here, in our investigation, RIP assay confirmed the correlation between miR-423-5p and SNHG4. Meanwhile, we found that miR-423-5p was significantly decreased in SNL rat models. SNHG4 regulated miR-423-5p expression negatively. As exhibited, the loss of miR-423-5p contributed to neuropathic pain progression, which was rescued by the silence of SNHG4. Therefore, our study indicated SNHG4 as a novel therapeutic target for neuropathic pain via sponging miR-423-5p.

## 1. Introduction

Neuropathic pain can result from the damage of neuronal tissues or a dysfunction of the nervous system [1]. It can be characterized by spontaneous pain, allodynia, and hyperalgesia [2]. The incidence of neuropathic pain in patients is increasing each year [3]. However, the pathophysiological mechanisms of the occurrence and development of neuropathic pain therapies are still barely known. Diagnosing and treating neuropathic pain is still a big clinical challenge.

lncRNAs are a family of long noncoding RNAs, which is characterized with transcripts with more than 200 nucleotides in molecular length [4–6]. Previous studies have pointed out that lncRNAs are involved in the progression of neuropathic pain [7–9]. For example, knockdown of lncRNA NONRATT021972 can inhibit diabetic neuropathic pain, which can be mediated by P2X3 receptor [10]. lncRNA X-inactive specific transcript can promote neuropathic pain development via modulating miR-154-5p and TLR5 in CCI rats [11]. XIST can induce neuropathic pain by sponging miR-544 and activating STAT3 [12]. The loss of lncRNA PKIA-AS1 can attenuate neuropathic pain via downregulating CDK6 [13]. SNHG4 has been identified in several physiological and pathological processes, participating in cancers. For example, upregulation of SNHG4 facilitates prostate cancer progression through regulating miR-377 and ZIC5 [14]. SNHG11 induces liver cancer progression via regulating miR-184 and AGO2 [15]. Nevertheless, the function and molecular mechanism of SNHG4 in neuropathic pain remain uninvestigated.

MicroRNAs are small noncoding RNAs which can exert a critical role in gene regulation [16]. MicroRNAs can modulate gene expression posttranscriptionally via binding to the protein-coding mRNAs with the corresponding complementary sequences [17]. Emerging roles of microRNAs in neuropathic pain have been exhibited [18, 19]. For instance, miR-142-3p can relieve neuropathic pain via regulating high mobility group box 1 [20]. miR-93 can alleviate neuropathic pain via targeting signal transducer and activator of transcription 3 [21]. The dysregulation of miR-423-5p is common in several diseases. For instance, miR-423-5p has been reported to inhibit high-glucose-induced podocyte injury through targeting Nox4 [22]. miR-423-5p can restrain myoblast proliferation and differentiation through targeting Sufu [23]. However, the function of miR-423-5p in neuropathic pain is still unexplored.

Here, we identified that SNHG4 in spinal cord tissue of SNL rats was greatly increased. The role of SNHG4 in SNL-triggered neuropathic pain was concentrated on. We observed that SNHG4 knockdown repressed neuropathic pain progression through inhibiting the neuroinflammation in vivo. By using bioinformatics analysis, we also found that overexpression of SNHG4 promoted neuropathic pain by directly sponging miR-423-5p. Therefore, our study indicated SNHG4 as a novel therapeutic target for neuropathic pain via regulating miR-423-5p.

## 2. Materials and Methods

### 2.1. Animal Studies

Male Sprague Dawley (SD) rats (∼300 g) were obtained from Shanghai Animal Laboratory Centre. We housed the rats in a facility kept in a standard 12-hour light/dark cycle at 24 ± 1°C and 50-70% humidity. The rats were provided with free access to water and food. Before the behavioral test, animals were housed for 1-2 days. Spinal nerve ligation- (SNL-) triggered neuropathic pain model was established as follows. After the rats were anesthetized using 1% sodium pentobarbital, L5 spinal nerves were isolated and ligated using 6-0 silk suture. In the sham-operated group, identical surgical procedure was carried out in the rats without ligation. Then, rats in each group were sacrificed following surgery for days 0, 3, 6, 9, and 15; the spinal cord tissue was collected immediately and maintained in -80°C for future experiment. The study was performed according to the requirements under the Guide for the Care and Use of Laboratory Animals of the NIH. This study was approved by the ethic committee of Renmin Hospital of Wuhan University.

### 2.2. Cell Culture

PC12 cells were obtained from ATCC (Manassas, VA, USA). Cells were cultured in DMEM medium (Invitrogen; Thermo Fisher Scientific, Inc., Waltham, MA, USA) with 10% FBS (Invitrogen, Carlsbad, CA, USA) and 1% penicillin/streptomycin (Invitrogen, Carlsbad, CA, USA). A 5% CO_2_ incubator at 37°C was used to incubate the cells. The cells were infected with the lentivirus (MOI = 50) with 5 mg/ml polybrene (Sigma-Aldrich, St. Louis, MO, USA) for 48 hours.

### 2.3. Lentivirus Infection

LV-anti-miR-435-5p, LV-shSNHG4, or their corresponding negative control LV-NCs were obtained from System Biosciences Inc. (Mountain View, CA, USA). SNL rats received intrathecal injections of either lentivirus (MOI = 100) on the third day after surgery. Intrathecal injection process was performed as follows. Rats were maintained in a prone location after anesthesia. A small opening at the intervertebral space between L4 and L6 vertebrae was created. Afterwards, a sterile PE10 intrathecal catheter was put via opening into the lumbar enlargement. Rats were allowed to recover for two days before other experiments were carried out, after receiving catheterization.

### 2.4. Assessment of Pain Threshold

Mechanical allodynia was assessed using paw withdrawal threshold (PWT) by von Frey filaments. Briefly, rats were put into a transparent plastic box with a metal mesh floor. We used the calibrated von Frey filaments (IITC, Woodland Hills, CA, United States) to create pressure on the plantar surface of rat hind paw. Then, the size of the filaments during paw withdrawal was recorded. Paw withdrawal latency (PWL) was utilized to assess the thermal hyperalgesia using the Plantar Test Instrument. The hind paws were recorded alternately at 5-minute intervals. The duration between stimuli and paw withdrawal was recorded. We set the cut-off time at 30 seconds.

### 2.5. RNA Immunoprecipitation Assay

Magna RIP Kit (EMD Millipore, Billerica, MA, USA) was used to do RNA immunoprecipitation assay according to the manufacturer's instruction. Cells were lysed using RIP lysis buffer. Magnetic beads conjugated to human anti-Ago2 antibody or control antibody (Millipore) were added to the cell lysate for a whole night.

### 2.6. ELISA

Spinal cord tissues were collected. Protein lysate was added to the spinal cord tissue samples of each group to homogenize the tissue. Then, we harvested the supernatant following centrifugation with 8,000 × g at 4°C. Levels of TNF-*α*, IL-12, IL-6, and IL-10 were detected using ELISA kits (Cwbiotech, Beijing, China). The concentrations of the standard wells were 0, 7.5, 15, 30, 60, and 120 pg/ml. In addition to the blank wells, 100 *μ*l HRP-labeled detection antibody (Abcam, Cambridge, USA) was added to the standard wells and sample wells. Afterwards, the wells were sealed by a sealing membrane, and following incubation at 37°C for one hour, the liquid was removed, the plate was dried, and the plate was repeatedly washed with PBS. 50 *μ*l of each of the substrates A and B was added to the well. The mixture was incubated at 37°C for 15 min without light. In the end, 50 *μ*l stop solution was added; the OD value of each well was determined using a microplate reader at 450 nm within 15 min. Protein concentration was calculated based on the standard curve.

### 2.7. Extraction of RNA and qRT-PCR

Total RNA from spinal cord tissues and microglias was extracted using TRIzol reagent (Invitrogen). To detect SNHG4 and miR-423-5p, M MLV reverse transcriptase (BioTeke Corp., Beijing, China) was used to obtain cDNA. qPCR was conducted using a SYBR-Green System (Applied Biosystems). Relative gene expression was exhibited using 2 *ΔΔ*Ct. Real-time PCR primer sequences were shown in Table 1. Primers for SNHG4, GAPDH, miR-423-5p, U6, IL-6, IL-12, IL-10, and TNF-*α* were obtained from Sangon (Shanghai, China).

### 2.8. RNA Pull-Down Assay

Purified RNAs were labeled using biotin and Pierce RNA 3′End Desthiobiotinylation Kit (Thermo Fisher Scientific, Waltham, MA, USA). Positive control (miR-423-5p-Bio), negative control (miR-423-5p-Bio-mut), and NC-Bio were incubated with the cell lysates. Each binding reaction was indicated with the magnetic beads. Eluted RNA was analyzed using qRT-PCR.

### 2.9. Statistical Analysis

Statistical analyses were carried out using GraphPad Prism software (GraphPad Software Inc., La Jolla, CA, USA). Student's *t*-test and one-way ANOVA were used to evaluate the statistical significance of the differences between two groups and multiple groups, respectively. *P* < 0.05 was considered statistically significant.

## 3. Results

### 3.1. A SNL Rat Model Was Successfully Established

**Figure 1:**
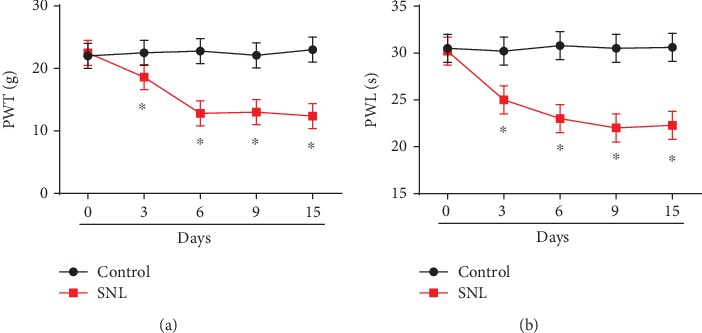
Spinal never ligation (SNL) rat models. Spinal never ligation induced (a) mechanical hyperalgesia and (b) thermal hyperalgesia in rats. *N* = 10 for each group; three independent experiments were carried out. Error bars stand for the mean ± SD of at least triplicate experiments. ∗*P* < 0.05.

Firstly, to investigate the role of SNHG4 in neuropathic pain triggered by spinal cord injury, a SNL rat model was established by us (Figures 1(a) and 1(b)). We evaluated thermal hyperalgesia and mechanical allodynia. In SNL rat models, PWT and PWL were greatly decreased at postoperative days 3, 6, 9, and 15.

### 3.2. SNHG4 Was Upregulated in SNL Rats

**Figure 2:**
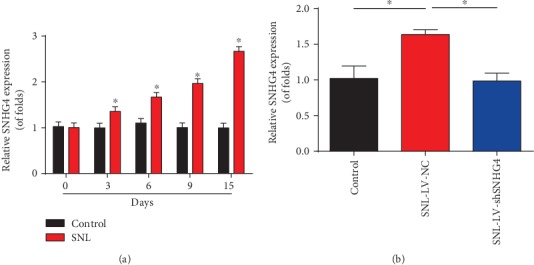
Expression of SNHG4 in SNL rats. (a) SNHG4 expression in the L4-L6 dorsal spinal cord of rats. qRT-PCR was used to detect SNHG4 expression at postoperative days 0, 3, 6, 9, and 15. (b) SNHG4 expression in rat models infected with LV-shSNHG4 or LV-NC at postoperative day 6. *N* = 10 in each group. Three independent experiments were carried out. Error bars stand for the mean ± SD of at least triplicate experiments. ∗*P* < 0.05.

To investigate the potential role of SNHG4 in neuropathic pain progression, RT-qPCR was employed to test SNHG4 expression in SNL rats. SNHG4 was obviously elevated in SNL rats compared to the control group at postoperative days 3, 6, 9, and 15 (Figure 2(a)). Subsequently, we conducted the loss-of-function experiments via intrathecal injection of LV shSNHG4. Infection with LV shSNHG4 greatly restrained SNHG4 expression in SNL rats as exhibited in Figure 2(b).

### 3.3. Loss of SNHG4 Relieved Neuropathic Pain

**Figure 3:**
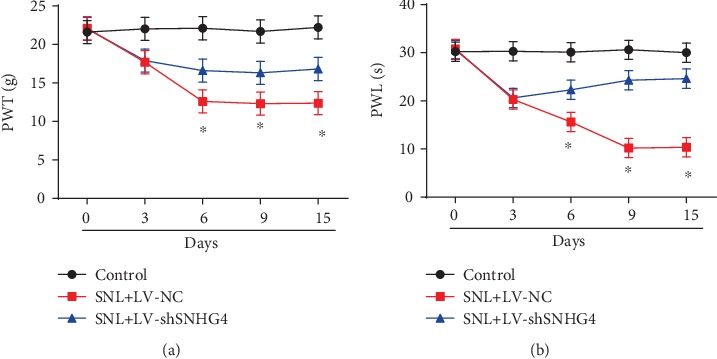
Knockdown of SNHG4 alleviated neuropathic pain. (a) The effect of LV-shSNHG4 on mechanical allodynia was evaluated by PWT. (b) The effect of LV-shSNHG4 on thermal hyperalgesia was assessed by PWL. *N* = 10 for each group. Three independent experiments were carried out. Error bars stand for the mean ± SD of at least triplicate experiments. ∗*P* < 0.05.

Furthermore, we demonstrated that silencing SNHG4 increased PWT (Figure 3(a)) and PWL (Figure 3(b)) in LV shSNHG4-infected rats compared to the LV NC-infected rat group at postoperative days 3, 6, 9, and 15.

### 3.4. Neuroinflammation Was Restrained by the Downregulation of SNHG4

**Figure 4:**
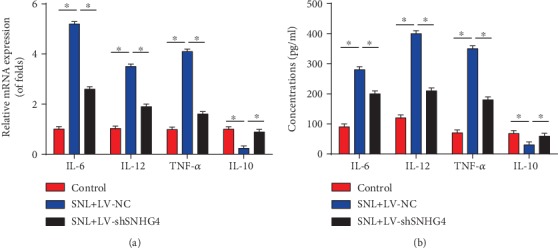
Silence of SNHG4 repressed neuroinflammation in SNL rats. (a) mRNA levels of IL-6, IL-12, TNF-*α*, and IL-10 in the L4-L6 dorsal spinal cord of rats were measured using qRT-PCR at postoperative day 6. (b) Protein levels of IL-6, IL-12, TNF-*α*, and IL-10 in the L4-L6 dorsal spinal cord of rats were measured using ELISA at postoperative day 6. *N* = 10 for each group. Three independent experiments were carried out. Error bars stand for the mean ± SD of at least triplicate experiments. ∗*P* < 0.05.

Moreover, to further explore the biological function of SNHG4, the effect of SNHG4 on neuroinflammation was evaluated by detecting IL-6, IL-12, TNF-*α*, and IL-10. The qRT-PCR data exhibited that the knockdown of SNHG4 inhibited IL-6, IL-12, and TNF-*α* mRNA expression while it increased the IL-10 level (Figure 4(a)). For another, IL-6, IL-12, and TNF-*α* protein expression was also inhibited by the loss of SNHG4 whereas IL-10 was induced by the knockdown of SNHG4 (Figure 4(b)).

### 3.5. miR-423-5p Was a Target for SNHG4

**Figure 5:**
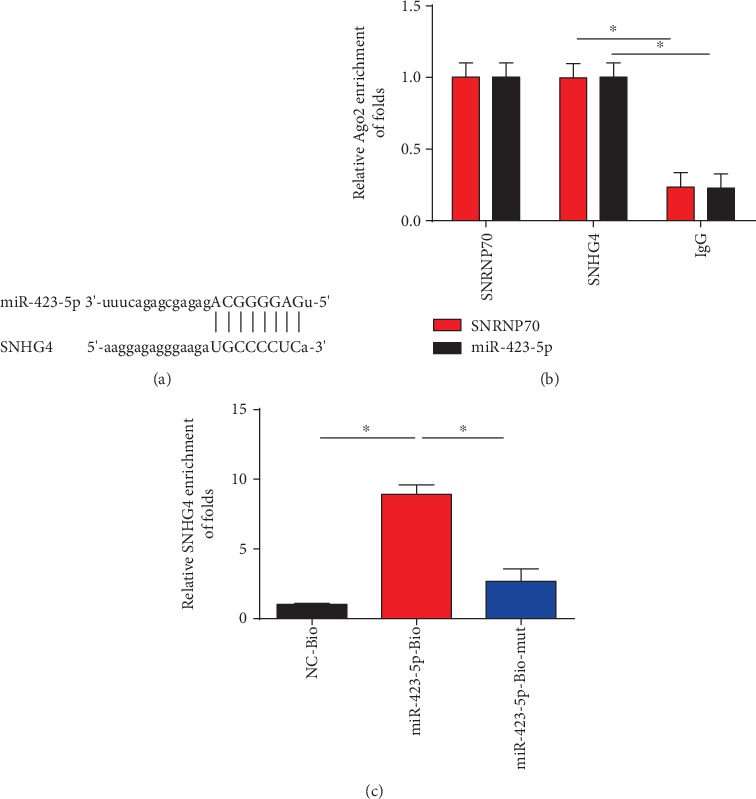
miR-423-5p was a target for SNHG4. (a) Binding regions between miR-423-5p and SNHG4. StarBase was used to predict the target microRNA regulated by SNHG4. (b) The correlation between SNHG4 and Ago2 was evaluated by RIP assay in PC12 cells. Cellular lysates were immunoprecipitated using Ago2 antibody or IgG. SNRNP70 level was employed as a positive control. (c) RNA pull-down assay indicated the direct interaction between miR-423-5p and SNHG4 in PC12 cells. Cellular lysates were pulled down using biotinylated control (NC-Bio), miR-423-5p (miR-423-5p-Bio), or miR-423-5p probe containing mutations in the SNHG4-binding site (miR-423-5p-Bio-mut). Three independent experiments were carried out. Error bars stand for the mean ± SD of at least triplicate experiments. ∗*P* < 0.05.

Bioinformatics analysis method (http://starbase.sysu.edu.cn/) was consulted by us to predict miR-423-5p as the target for SNHG4 (Figure 5(a)). Additionally, RIP assay was carried out in our study and we confirmed the direct correlation between them (Figure 5(b)). As exhibited, SNHG4 and miR-423-5p were much more abundant in Ago2 pellet than in IgG pellet in PC12 cells. RNA pull-down assay also validated that SNHG4 could directly target miR-423-5p (Figure 5(c)). Biotinylated miR-423-5p (miR-423-5p-Bio) probe increased the level of SNHG4 than control (NC-bio) or miR-423-5p probes.

### 3.6. miR-423-5p Was Downregulated in SNL Rats

**Figure 6:**
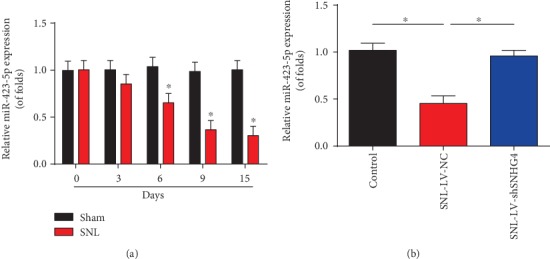
Expression of miR-423-5p in SNL rats. (a) miR-423-5p expression in the L4-L6 dorsal spinal cord of rats. qRT-PCR was used to detect SNHG4 expression at postoperative days 0, 3, 6, 9, and 15. (b) miR-423-5p expression in rat models infected with LV-shSNHG4 or LV-NC at postoperative day 6. *N* = 10 in each group. Three independent experiments were carried out. Error bars stand for the mean ± SD of at least triplicate experiments. ∗*P* < 0.05.

In addition, we observed that miR-423-5p was strongly downregulated in SNL rats compared to the control group (Figure 6(a)). Then, as indicated in Figure 6(b), LV shSNHG4 greatly elevated miR-423-5p expression in SNL rats.

### 3.7. SNHG4 Reversed the Effect of miR-423-5p on Neuropathic Pain

**Figure 7:**
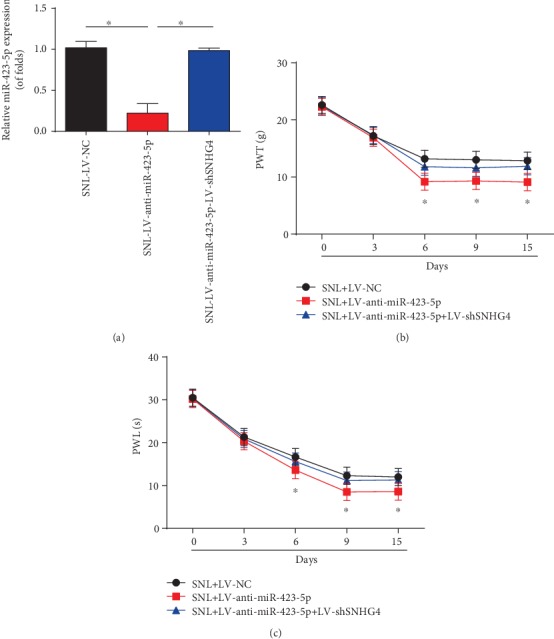
SNHG4 reversed the effect of miR-423-5p on neuropathic pain. (a) miR-423-5p expression in the L4-L6 dorsal spinal cord of rats infected with LV-anti-miR-423-5p or LV-anti-miR-423-5p and LV-shSNHG4 at postoperative day 6. (b) The effect of miR-423-5p on mechanical allodynia was evaluated by PWT. (c) The effect of miR-423-5p on thermal hyperalgesia was assessed by PWL. *N* = 10 for each group. Three independent experiments were carried out. Error bars stand for the mean ± SD of at least triplicate experiments. ∗*P* < 0.05.

We examined the effect of miR-423-5p on neuropathic pain. miR-423-5p was greatly inhibited by LV-anti-miR-423-5p, which was reversed by the loss of SNHG4 (Figure 7(a)). In addition, we displayed that inhibition of miR-423-5p decreased PWT (Figure 7(b)) and PWL (Figure 7(c)) at postoperative days 3, 6, 9, and 15, which was rescued by the knockdown of SNHG4.

### 3.8. SNHG4 Reversed the Effect of miR-423-5p on Neuroinflammation

**Figure 8:**
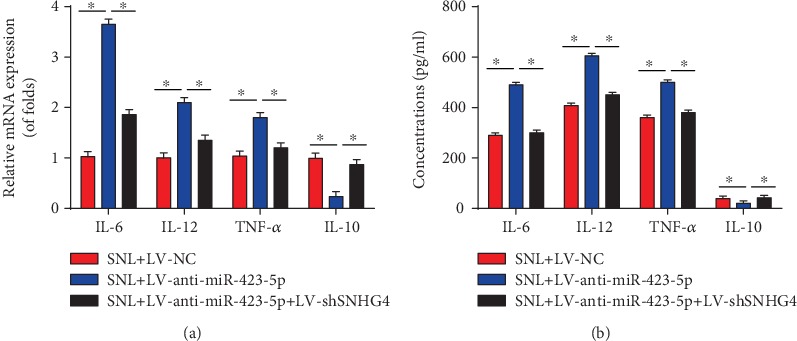
SNHG4 reversed the effect of miR-423-5p on neuroinflammation in SNL rats. (a) mRNA levels of IL-6, IL-12, TNF-*α*, and IL-10 in the L4-L6 dorsal spinal cord of rats were measured using qRT-PCR at postoperative day 6. (b) Protein levels of IL-6, IL-12, TNF-*α*, and IL-10 in the L4-L6 dorsal spinal cord of rats were measured using ELISA at postoperative day 6. *N* = 10 for each group. Three independent experiments were carried out. Error bars stand for the mean ± SD of at least triplicate experiments. ∗*P* < 0.05.

The effect of miR-423-5p on neuroinflammation was evaluated. We found that the knockdown of miR-423-5p could increase IL-6, IL-12, and TNF-*α* mRNA expression and decrease IL-10 mRNA level, which could be reversed by the loss of SNHG4 (Figure 8(a)). In addition, IL-6, IL-12, and TNF-*α* protein expression was restrained by the loss of miR-423-5p whereas IL-10 was elevated by miR-423-5p inhibition (Figure 8(b)), which was totally reverted by the knockdown of SNHG4.

## 4. Discussion

Neuropathic pain is a problem for patients following spinal surgery, and it is associated with abnormal sensations [1, 2]. Although great progress has been made in the treatment, a number of patients still suffer chronic pain. The molecular mechanism of neuropathic pain is still to be elucidated. Recently, many noncoding RNAs have been reported in neuropathic pain progression [24]. Currently, we indicated that SNHG4 was upregulated in SNL model rats. The present data proved that SNHG4 contributed to neuropathic pain progression via triggering the neuroinflammation. In addition, miR-423-5p was predicted as the target of SNHG4 and we found that miR-423-5p was obviously decreased in the rats. Upregulation of SNHG4 reversed the effect of miR-423-5p on neuropathic pain.

Excessive activation of spinal dorsal horn neurons after nerve injury contributes to the development of neuropathic pain [25, 26]. Activation of microglia and astrocytes can cause subsequent overproduction of inflammatory mediators such as TNF-*α*, IL-1*β*, and IL-6 [27, 28]. In addition, inhibition of microglial activation or astrocytic activation can attenuate neuropathic pain progression effectively [29, 30].

Previously, it has been shown that lncRNA SNHG4 can promote osteosarcoma growth via sponging miR-224-3p, which can predict a poor survival and recurrence [31]. In addition, SNHG4 can promote cervical cancer progression via regulating miR-206 and YWHAZ [32]. These indicate that SNHG4 exert a crucial role in various cancers. Currently, we found SNHG4 was increased in SNL rat models. These indicated that SNHG4 might serve a role in neuropathic pain. Then, to further investigate the function of SNHG4 in the neural system of SNL rats, LV-shSNHG4 lentivirus was administered using intrathecal injection. We found that the knockdown of SNHG4 repressed neuropathic pain progression. The neuroinflammation was strongly suppressed by the loss of SNHG4. IL-6, IL-12, and TNF-*α* were inhibited while IL-10 was induced by the inhibition of SNHG4.

Via using bioinformatics analysis, miR-423-5p was predicted as the target of SNHG4. miR-423-5p has been reported as a significant regulator in various diseases. For example, miR-423-5p is involved in lupus nephritis via regulating the activation of NF-*κ*B and targeting TNIP2 [33]. miR-423-5p can contribute to temozolomide chemoresistance of glioblastomas [34]. Knockdown of miR-423-5p can enhance glioma stem cells sensitivity to apigenin via regulating the mitochondrial pathway [35]. In ovarian cancer, miR-423-5p can serve as an indicator, which can repress proliferation and invasion [36]. In addition, lncRNA AFAP1-AS1 can act as a ceRNA of miR-423-5p, which can facilitate nasopharyngeal carcinoma by regulating Rho/Rac pathway [37]. Here, we observed that miR-423-5p was decreased in SNL models. miR-423-5p was negatively regulated by SNHG4, and the direct correlation between them was validated in our investigation. Additionally, the loss of SNHG4 was able to reverse the effect of miR-423-5p inhibition on neuroinflammation and neuropathic pain. These data demonstrated that SNHG4 could directly bind with miR-423-5p. However, the roles of miR-154-5p in CCI rats remained unclear. Then, based on the bioinformatics method (http://starbase.sysu.edu.cn/), so many mRNA transcripts have been predicted. Some mRNA transcripts have been reported to participate in neuropathic pain development, such as STAT3 and HMGA2. For example, overexpression of miR-98 reduces neuropathic pain by targeting STAT3 [38]. miR-93 alleviates neuropathic pain via targeting STAT3 [35]. In addition, downregulation of HMGA2 by miR-98 represses neuropathic pain [39]. In our future study, we would like to do more investigations to clarify the potential mechanism of miR-423-5p in neuropathic pain. Taken together, the present study demonstrated that the inhibition of SNHG4 in SNL rats could reduce neuropathic pain via repressing neuroinflammation. This suggested that SNHG4 might serve as a new target for treating neuropathic pain in humans, which can provide a new intervention strategy for neuropathic pain patients. However, this should be well verified in patients.

## 5. Conclusions

In conclusion, we revealed that SNHG4 induced neuropathic pain progression via sponging miR-423-5p in SNL model rats.

## Figures and Tables

**Table 1:** Primers used for real-time PCR.

Genes	Forward (5′-3′)	Reverse (5′-3′)
GAPDH	CAAGGTCATCCATGACAACTTTG	GTCCACCACCCTGTTGCTGTAG
U6	CTCGCTTCGGCAGCACA	AACGCTTCACGAATTTGCGT
SNHG4	GCAGGTGACAGTCTGCATGT	TTTTAAGTCCCCTACCCCCATC
miR-423-5p	GGGGTGAGGGGCAGAGAG	TGCGTGTCGTGGAGTC
IL-6	GACTGATGTTGTTGACAGCCACTGC	AGCCACTCCTTCTGTGACTCTAACT
IL-12	GGACATCATCAAACCTGACC	AGGGAGAAGTAGGAATGTGG
IL-10	CCAGTCTGAGAACAGCTGCA	AGCCCCAGATCCGATTTTGG
TNF-*α*	CATGATCCGAGATGTGGAACTGGC	CTGGCTCAGCCACTCCAGC

## Data Availability

The data used to support the findings of this study are available from the corresponding author upon request.

## Abbreviations

SNHG4: Small Nucleolar RNA Host Gene 4
lncRNAs: Long noncoding RNAs
TNF-*α*: Tumor necrosis factor-*α*
IL: Interleukin
SNL: Spinal nerve ligation
ELISA: Enzyme-linked immunosorbent assay
qRT-PCR: Quantitative real-time polymerase chain reaction
CCK-8: Cell Counting Kit-8
EdU: 5-Ethynyl-2′-deoxyuridine
RIP: RNA immunoprecipitation assay.
