# Corrigendum to “Loss of SNHG4 Attenuated Spinal Nerve Ligation-Triggered Neuropathic Pain through Sponging miR-423-5p”

**DOI:** 10.1155/2020/6369898

**Published:** 2020-10-14

**Authors:** Xia Pan, Cheng Shen, Yayi Huang, Long Wang, Zhongyuan Xia

**Affiliations:** Department of Anesthesia, Renmin Hospital of Wuhan University, Wuhan Hubei, China

In the article titled “Loss of SNHG4 Attenuated Spinal Nerve Ligation-Triggered Neuropathic Pain through Sponging miR-423-5p” [1], the last sentence in Results (Section 3.8) contains an error, where the text reading “In addition, IL-6, IL-12, and TNF-*α* protein expression was restrained by the loss of miR-423-5p whereas IL-10 was elevated by miR-423-5p inhibition (Figure 8(b)), which was totally reverted by the knockdown of SNHG4.” should be corrected to “In addition, IL-6, IL-12, and TNF-*α* protein expression was induced by the loss of miR-423-5p whereas IL-10 was reduced by miR-423-5p inhibition (Figure 8(b)), which was totally reverted by the knockdown of SNHG4.”
